# Supplementary material for: Genotype and growth rate influence female mate preference in Xiphophorus multilineatus: Potential selection to optimize mortality-growth rate tradeoff
Source: PLoS One. 2023 Jun 29;18(6):e0287843. doi: 10.1371/journal.pone.0287843 (PMC10310027; doi:10.1371/journal.pone.0287843)
Supplement: S1 Dataset — (PDF) [file pone.0287843.s001.pdf]

Experiment_1

Female_new_ID	femaleID	SL	Mesocosm	Mescosm Type	MalePairID	Replicate	SneakerID	sneakerSL	CourterID	courterSL	Time Spent with Courter	Time spent with sneaker	Stregth of Preference	MaleSizeDiff	Association
1	11	30.41	Courter	1	S1C1	a	S1	27.19	C1	35.19	775	349	426	8	1124
2	12	33.08	Courter	1	S2C2	a	S2	27.44	C2	36.44	559	510	49	9	1069
3	13	30.92	Courter	1	S3C3	a	S3	28.78	C3	33.09	694	354	340	4.31	1048
4	14	29.7	Courter	1	S4C4	a	S4	27.23	C4	32.89	352	680	-328	5.66	1032
5	15	34.35	Courter	1	S5C5	a	S5	27.88	C5	35.67	584	593	-9	7.79	1177
6	16	35.15	Courter	1	S1C2	a	S1	27.19	C2	36.44	654	492	162	9.25	1146
7	17	32.53	Courter	1	S2C3	a	S2	27.44	C3	33.09	0	572	-572	5.65	572
8	18	32.93	Courter	1	S3C4	a	S3	28.78	C4	32.89	326	584	-258	4.11	910
9	19	31.44	Courter	1	S4C5	a	S4	27.23	C5	35.67	978	91	887	8.44	1069
10	20	35.76	Courter	1	S5C1	a	S5	27.88	C1	35.19	666	490	176	7.31	1156
11	40	26.58	Courter	1	S5C1	a	S5	27.88	C1	35.19	480	634	-154	7.31	1114
12	41	28.85	Courter	1	S1C5	a	S1	27.19	C5	35.67	488	443	45	8.48	931
39	1	41.74	Courter	1	S6C6	b	S6	27.6	C6	35.33	592	525	67	7.73	1117
40	3	32.02	Courter	1	S7C7	b	S7	27.7	C7	35.87	405	644	-239	8.17	1049
41	4	31.99	Courter	1	S8C8	b	S8	26.32	C8	33.6	356	691	-335	7.28	1047
42	5	42.57	Courter	1	S9C9	b	S9	28.5	C9	35.88	301	709	-408	7.38	1010
43	6	30.7	Courter	1	S10C6	b	S10	28.22	C6	35.33	484	581	-97	7.11	1065
44	8	33.02	Courter	1	S8C7	b	S8	26.32	C7	35.87	463	620	-157	9.55	1083
45	9	30.35	Courter	1	S9C8	b	S9	28.5	C8	33.6	747	347	400	5.11	1094
46	10	33.68	Courter	1	S6C9	b	S6	27.6	C9	35.88	332	367	-35	8.28	699
47	11	35.35	Courter	1	S7C6	b	S7	27.7	C6	35.33	602	500	102	7.62	1102
48	12	35.39	Courter	1	S8C10	b	S8	26.32	C10	34.39	1126	0	1126	8.06	1126
49	13	32.8	Courter	1	S9C7	b	S9	28.5	C7	35.87	637	484	153	7.38	1121
50	14	34.49	Courter	1	S6C8	b	S6	27.6	C8	33.6	852	252	600	6.01	1104
51	16	31.66	Courter	1	S8C6	b	S8	26.32	C6	35.33	1069	88	981	9	1157
52	17	30.37	Courter	1	S9C10	b	S9	28.5	C10	34.39	405	701	-296	5.89	1106
53	18	30.55	Courter	1	S6C7	b	S6	27.6	C7	35.87	848	245	603	8.28	1093
54	19	42.34	Courter	1	S10C8	b	S10	28.22	C8	33.6	602	468	134	5.39	1070
55	20	30.31	Courter	1	S7C9	b	S7	27.7	C9	35.88	451	624	-173	8.17	1075
56	31	31.83	Courter	1	S7C10	b	S7	27.7	C10	34.39	646	99	547	6.68	745
57	32	33.85	Courter	1	S10C9	b	S10	28.22	C9	35.88	641	364	277	7.66	1005
58	43	30.34	Courter	1	S10C10	b	S10	28.22	C10	34.39	588	584	4	6.17	1172
13	10	29.91	Mixed	2	S5C5	a	S5	27.88	C5	35.67	871	170	701	7.79	1041
14	2	34.23	Mixed	2	S2C1	a	S2	27.44	C1	35.19	565	523	42	7.75	1088
15	32	25.5	Mixed	2	S2C2	a	S2	27.44	C2	36.44	1039	101	938	9	1140
16	35	25.03	Mixed	2	S5C5	a	S5	27.88	C5	35.67	1087	61	1026	7.79	1148
17	3	27.68	Mixed	2	S3C2	a	S3	28.78	C2	36.44	344	649	-305	7.66	993
18	5	38.37	Mixed	2	S5C4	a	S5	27.88	C4	32.89	221	899	-678	5.01	1120
19	6	29.39	Mixed	2	S1C1	a	S1	27.19	C1	35.19	1172	0	1172	8	1172
20	8	32.47	Mixed	2	S3C3	a	S3	28.78	C3	33.09	794	203	591	4.31	997
21	9	30.01	Mixed	2	S4C4	a	S4	27.23	C4	32.89	344	603	-259	5.66	947
79	54	29.36	Mixed	2	S6C6	b	S6	27.6	C6	35.33	639	370	269	7.73	1009
80	55	24.99	Mixed	2	S10C10	b	S10	28.22	C10	34.39	526	460	66	6.17	986
81	56	29.01	Mixed	2	S7C8	b	S7	27.7	C8	33.6	762	326	436	5.9	1088
82	57	29.55	Mixed	2	S8C6	b	S8	26.32	C6	35.33	644	391	253	9	1035
83	58	27.49	Mixed	2	S9C9	b	S9	28.5	C9	35.88	139	655	-516	7.38	794
84	59	24.95	Mixed	2	S9C7	b	S9	28.5	C7	35.87	623	516	107	7.38	1139
85	60	27.7	Mixed	2	S6C8	b	S6	27.6	C8	33.6	371	689	-318	6.01	1060
86	61	26.72	Mixed	2	S9C8	b	S9	28.5	C8	33.6	141	945	-804	5.11	1086
87	62	26.35	Mixed	2	S6C9	b	S6	27.6	C9	35.88	65	936	-871	8.28	1001
22	23	32.4	Sneaker	1	S3C4	a	S3	28.78	C4	32.89	1159	0	1159	4.11	1159
23	24	40.53	Sneaker	1	S4C5	a	S4	27.23	C5	35.67	498	295	203	8.44	793
24	25	36.63	Sneaker	1	S5C1	a	S5	27.88	C1	35.19	855	184	671	7.31	1039
25	27	32.77	Sneaker	1	S2C1	a	S2	27.44	C1	35.19	502	531	-29	7.75	1033
26	28	33.31	Sneaker	1	S3C2	a	S3	28.78	C2	36.44	304	764	-460	7.66	1068
27	29	31.86	Sneaker	1	S4C3	a	S4	27.23	C3	33.09	1545	0	1545	5.86	1545
28	30	28.68	Sneaker	1	S5C4	a	S5	27.88	C4	32.89	545	289	256	5.01	834
29	33	39.16	Sneaker	1	S3C3	a	S3	28.78	C3	33.09	727	79	648	4.31	806
30	34	35.28	Sneaker	1	S4C4	a	S4	27.23	C4	32.89	660	342	318	5.66	1002
31	37	25.83	Sneaker	1	S2C3	a	S2	27.44	C3	33.09	912	198	714	5.65	1110
32	38	39.19	Sneaker	1	S3C4	a	S3	28.78	C4	32.89	474	106	368	4.11	580
33	39	33.94	Sneaker	1	S4C5	a	S4	27.23	C5	35.67	717	37	680	8.44	754
34	42	32.55	Sneaker	1	S2C1	a	S2	27.44	C1	35.19	828	209	619	7.75	1037
35	43	31.51	Sneaker	1	S3C2	a	S3	28.78	C2	36.44	663	384	279	7.66	1047
36	45	36.68	Sneaker	1	S5C4	a	S5	27.88	C4	32.89	874	296	578	5.01	1170
37	46	37.94	Sneaker	1	S1C1	a	S1	27.19	C1	35.19	769	375	394	8	1144
38	47	38.88	Sneaker	1	S2C2	a	S2	27.44	C2	36.44	142	269	-127	9	411
59	21	40.2	Sneaker	1	S6C6	b	S6	27.6	C6	35.33	485	533	-48	7.73	1018
60	22	34.65	Sneaker	1	S10C10	b	S10	28.22	C10	34.39	913	161	752	6.17	1074
61	23	30.71	Sneaker	1	S7C7	b	S7	27.7	C7	35.87	1030	53	977	8.17	1083
62	24	33.64	Sneaker	1	S8C8	b	S8	26.32	C8	33.6	427	486	-59	7.28	913
63	25	27.26	Sneaker	1	S9C9	b	S9	28.5	C9	35.88	1086	8	1078	7.38	1094
64	26	32.59	Sneaker	1	S10C6	b	S10	28.22	C6	35.33	819	302	517	7.11	1121
65	27	32.91	Sneaker	1	S7C10	b	S7	27.7	C10	34.39	626	527	99	6.68	1153
66	28	28.61	Sneaker	1	S8C7	b	S8	26.32	C7	35.87	1126	11	1115	9.55	1137
67	29	27.23	Sneaker	1	S9C8	b	S9	28.5	C8	33.6	664	439	225	5.11	1103
68	30	35.66	Sneaker	1	S6C9	b	S6	27.6	C9	35.88	746	4	742	8.28	750
69	44	34.58	Sneaker	1	S7C6	b	S7	27.7	C6	35.33	1049	43	1006	7.62	1092

**70**	45	32.97	Sneaker	1	S8C10	b	S8	26.32	C10	34.39	413	614	-201	8.06	1027
**71**	46	29.47	Sneaker	1	S9C7	b	S9	28.5	C7	35.87	837	230	607	7.38	1067
**72**	47	33.6	Sneaker	1	S6C8	b	S6	27.6	C8	33.6	867	197	670	6.01	1064
**73**	48	35.43	Sneaker	1	S10C9	b	S10	28.22	C9	35.88	763	318	445	7.66	1081
**74**	49	35.89	Sneaker	1	S8C6	b	S8	26.32	C6	35.33	942	179	763	9	1121
**75**	50	34.73	Sneaker	1	S9C10	b	S9	28.5	C10	34.39	172	835	-663	5.89	1007
**76**	51	32.76	Sneaker	1	S6C7	b	S6	27.6	C7	35.87	424	667	-243	8.28	1091
**77**	52	32.02	Sneaker	1	S10C8	b	S10	28.22	C8	33.6	661	441	220	5.39	1102
**78**	53	40.77	Sneaker	1	S7C9	b	S7	27.7	C9	35.88	914	93	821	8.17	1007
