# Supplementary material for: Genotype and growth rate influence female mate preference in Xiphophorus multilineatus: Potential selection to optimize mortality-growth rate tradeoff
Source: PLoS One. 2023 Jun 29;18(6):e0287843. doi: 10.1371/journal.pone.0287843 (PMC10310027; doi:10.1371/journal.pone.0287843)
Supplement: S2 Dataset — (PDF) [file pone.0287843.s002.pdf]

Experiment_2

FemaleID	Lineage	LineageScored	FirstSL	SecondSL	Growth_Rate	Stregth of Preference Virgin	IDcourter1	Courter_SL_1	IDSneaker1	SneakerSL1	SizeDiff1	Stregth of Preference Exp	IDCourter2	CourterSL2	IDSenaker2	SneakerSL2	SizeDiff2	Delta_SOP	Delta_Courter
**F2**	Courter	1	28.65	39.62	0.036	809	C3	35.93	S3	28.41	7.52	282	C3	35.93	S3	28.41	7.52	527	0
**F4**	Sneaker	2	33.15	37.79	0.015	249	C5	33.63	S3	28.41	5.22	1102	C1	36.5	S5	28.46	8.04	-853	-2.87
**F5**	Sneaker	2	31.65	36.79	0.017	943	C5	33.63	S4	27.19	6.44	605	C4	33.94	S3	28.41	5.53	338	-0.31
**F6**	Courter	1	30.38	38.61	0.027	678	C1	36.5	S2	28.29	8.21	1073	C2	33.69	S1	27.91	5.78	-395	2.81
**F7**	Sneaker	2	29.95	39.57	0.032	1074	C2	33.69	S2	28.29	5.4	162	C3	35.93	S5	28.46	7.47	912	-2.24
**F8**	Courter	1	30.53	41.09	0.035	41	C1	36.5	S4	27.19	9.31	438	C3	35.93	S2	28.29	7.64	-397	0.57
**F11**	Courter	1	27.47	37.13	0.032	454	C3	35.93	S5	28.46	7.47	1040	C3	35.93	S4	27.19	8.74	-586	0
**F13**	Sneaker	2	31.1	35.75	0.015	588	C5	33.63	S4	27.19	6.44	1154	C3	35.93	S1	27.91	8.02	-566	-2.3
**F15**	Sneaker	2	32.92	39.97	0.023	-429	C5	33.63	S5	28.46	5.17	913	C3	35.93	S5	28.46	7.47	-1342	-2.3
**F17**	Courter	1	31.22	36.67	0.018	308	C1	36.5	S1	27.91	8.59	6	C1	36.5	S1	27.91	8.59	302	0
**F18**	Sneaker	2	33.14	39.55	0.021	1	C4	33.94	S4	27.19	6.75	539	C1	36.5	S2	28.29	8.21	-538	-2.56
**F21**	Sneaker	2	34.18	36.73	0.008	1171	C1	36.5	S5	28.46	8.04	1171	C4	33.94	S5	28.46	5.48	0	2.56
**F22**	Sneaker	2	34.03	39.21	0.017	840	C1	36.5	S3	28.41	8.09	371	C5	33.63	S4	27.19	6.44	469	2.87
**F23**	Courter	1	34.79	41.36	0.022	101	C2	33.69	S4	27.19	6.5	444	C1	36.5	S2	28.29	8.21	-343	-2.81
**F24**	Courter	1	34.27	40.67	0.021	504	C3	35.93	S1	27.91	8.02	547	C1	36.5	S3	28.41	8.09	-43	-0.57
**F26**	Courter	1	28.87	39.58	0.036	1000	C2	33.69	S2	28.29	5.4	994	C5	33.63	S5	28.46	5.17	6	0.06
**F28**	Sneaker	2	30.84	37.62	0.023	-264	C5	33.63	S2	28.29	5.34	1052	C2	33.69	S4	27.19	6.5	-1316	-0.06
**F29**	Sneaker	2	33.84	40.12	0.021	549	C2	33.69	S3	28.41	5.28	896	C4	33.94	S4	27.19	6.75	-347	-0.25
**F31**	Courter	1	33.31	39.52	0.021	354	C4	33.94	S3	28.41	5.53	664	C1	36.5	S1	27.91	8.59	-310	-2.56
**F32**	Courter	1	30.67	37.7	0.023	454	C1	36.5	S5	28.46	8.04	401	C4	33.94	S2	28.29	5.65	53	2.56
